# Age Distribution of Patients Presenting with Uveitis

**DOI:** 10.2174/1874364100701010023

**Published:** 2007-12-17

**Authors:** Holger Baatz, Gábor B Scharioth, Diego de Ortueta, Mitrofanis Pavlidis

**Affiliations:** Eye Centre Recklinghausen, Erlbruch 34-36, D-45657 Recklinghausen, Germany

## Abstract

**Background:**

Endogenous uveitis has long been considered to be uncommon in elderly patients. Recently, population-based studies have shown an increase in the incidence of uveitis with inreasing age. It was the aim of this study to analyse the age distribution of patients presenting with endogenous uveitis in the setting of an eye centre.

**Methods:**

Retrospective cohort analysis.

**Results:**

278 patients with endogenous uveitis showed the following age distribution: 0 - 15: 9, 15 - 30: 28, 30 - 45: 73, 45 - 60: 78, >60: 90 (age group: number of patients). Localization of uveitis was 82,4% anterior, 9,4% intermedia, 12,9% posterior. Two patients >60y were diagnosed with a masquerade syndrome.

**Conclusions:**

A large number of patients presenting with endogenous uveitis is in the elderly age group. An early and appropriate diagnostic work-up for uveitis is recommended for the elderly.

## INTRODUCTION

Endogenous uveitis is a potentially sight-threatening condition. Early diagnosis and initiation of treatment may reduce the risk of permanently reduced visual acuity. The differential diagnosis of uveitis may include a broad spectrum of ocular diseases, e.g. trauma, degenerative diseases, dystrophies, diabetic retinopathy or vascular occlusion. In the past decades, endogenous uveitis was considered to have a peak incidence in middle-aged individuals and to be less likely to occur in the elderly. This assumption was based on the epidemiological study by Darrell *et al*. [1], which for more than 40 years has been the only population-based epidemiological study on uveitis in the U.S. The findings of Darrell *et al*. have influenced the contents of many textbooks. Recently, two large population-based studies in the U.S. have challenged the concept that endogenous uveitis is most common in middle-aged persons. Rather, these sudies have shown an increased incidence of endogenous uveitis with increasing age [2, 3]. In light of these findings we have retrospectively evaluated patient data from our clinic. It was the aim of this study to analyze the age distribution of patients presenting with endogenous uveitis in our clinic, and thus to estimate the priority that should be awarded to a uveitis work-up for the differential diagnosis of endogenous uveitis in the elderly patient.

## METHODS

The electronic patient records from all patients presenting in our clinic between January, 1^st^ 2002 and March, 1^st^ 2007 were retrospectively searched for diagnoses indicating intraocular inflammation. Patient records are exclusively in the form of electronically stored data (ifa Systems, Cologne, Germany). As the ICD-10 code does not sufficiently differentiate between ophthalmological disease entities, diagnoses are also stored in text form. This eliminates the loss of information by unexact representation of diagnoses in the ICD-10. Data analysis comprised two steps: Firstly, a database search was done for all relevant diagnoses that were indicative of intraocular inflammation, e.g. cystoid macular edema. The search also included systemic diseases known to be associated with intraocular inflammation, e.g. sarcoidosis. Secondly, the individual patient records that came up in the first database search were reviewed individually. The classification of uveitis adhered to the Standardization of Uveitis Nomenclature (SUN) Working Group [4]. Only patients suffering from active endogenous uveitis were included. Standard workups for uveitis included a detailed patient history and anterior and posterior segment biomicroscopy. Thereafter, an organized diagnostic evaluation with laboratory testing and medical consultations were planned accordingly. Patients with diabetic retinopathy, thromboembolic diseases of retinal vessels and postoperative intraocular inflammation within three months of intraocular surgery were excluded. Patients with inactive inflammatory lesions of the choroid and / or retina, or residuals of past episodes of uveitis were also excluded, as long as there was no recurrence within the observation period (e.g. inactive toxoplasmosis scars).

## RESULTS

After reviewing the individual patient records, 278 patients were identified who presented with active endogenous uveitis between January, 1^st^ 2002 and March, 1^st^ 2007. The age distribution is presented in Fig. (**1**). The anatomical classification and gender distribution of uveitis patients is presented in Table **1**. In two patients who were over 60 years old, an intraocular lymphoma was diagnosed.

## CONCLUSIONS

The data show clearly that endogenous uveitis in elderly patients is not a rare diagnosis. The data are not population-based and therefore do not allow any conclusions about the prevalence or incidence of endogenous uveitis in the general population. However, the data does give an insight into the age distribution of patients presenting with endogenous uveitis. The data does support the findings of population-based studies which describe an increasing incidence of uveitis with increasing age [2, 3]. We specifically excluded patients with inactive lesions of uveitis that were found coincidentally and were not related to the patient´s complaints. This selection was made because determining the prevalence of (past episodes of) uveitis in a defined population was not an intenion of this study. The frequencies of anatomic distribution of uveitis is in line with that reported in the literature [5]. Only few studies have examined the characteristics of endogenous uveitis in elderly patients [5-9]. These findings are relevant for the differential diagnosis of elderly patients presenting with signs of intraocular inflammation. While masquerade syndromes most often affect the elderly, they are still rare even in patients over 60 years old and often pose a challenge for the ophthalmologist [10]. We have found two patients with a masquerade-syndrome, both were older than 60 years.

In conclusion, our study shows that in clinical practice, a diagnosis of endogenous uveitis is made at least as often in patients over 60 years of age as in the younger age groups. We therefore recommend an early and appropriate diagnostic work-up for uveitis in the elderly presenting with signs of intraocular inflammation.

## Figures and Tables

**Fig. (1):**
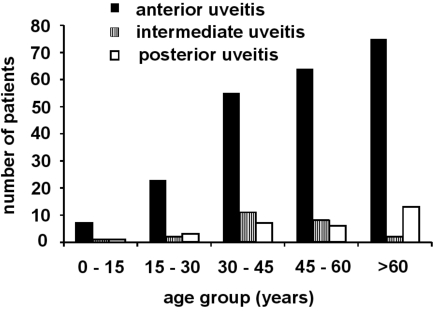
Age distribution of patients with endogenous uveitis (n = 278).

**Table 1.** Anatomic Localisation of Uveitis

Localisation		Patients Abs (%)
anterior	male	104 (37,4)
	female	125 (45,0)
	all	229 (82,4)
intermediate	male	10 (3,6)
	female	16 (5,8)
	all	26 (9,4)
posterior	male	19 (6,8)
	female	17 (6,1)
	all	36 (12,9)

Multiple counts possible if uveitis affects more than one segment of the eye and the predominant site of inflammation can not be determined.
